# Python-driven impedance profiling on peptide-functionalized biosensor for detection of HIV gp41 envelope protein

**DOI:** 10.1007/s13205-025-04400-8

**Published:** 2025-06-30

**Authors:** Mehmet Ezer, Zihni Onur Uygun

**Affiliations:** 1 Department of Urology, Faculty of Medicine, Kafkas University, 36100 Kars, Turkey; 2 Department of Medical Biochemistry, Faculty of Medicine, Kafkas University, 36100 Kars, Turkey

**Keywords:** Biosensor, HIV, Gp41, Impedance, Python

## Abstract

This study presents the first label-free impedimetric biosensor for the detection of HIV envelope protein gp41 using antimicrobial peptides (AMPs) as biorecognition receptors. The biosensor interface was enhanced with thiolated polystyrene and gold nanospheres to ensure stable peptide immobilization and resist nonspecific adsorption. Electrochemical impedance spectroscopy (EIS) confirmed each step of the electrode modification, while surface morphology was validated via scanning electron microscopy. A Python-based deep learning algorithm was applied to impedance data for efficient curve fitting and regression modeling. The biosensor demonstrated high sensitivity, with a linear detection range of 5–600 pg/mL, a regression coefficient (*R*^2^) of 0.9946, a limit of detection (LOD) of 1.62 pg/mL, and a limit of quantification (LOQ) of 4.91 pg/mL. Chronoimpedimetric (CI) detection revealed that gp41 binding occurred within 350 s. The biosensor showed excellent reproducibility (CV % = 0.22%), good selectivity with less than 12% signal variation in spiked serum, and robust stability, maintaining functionality after extended storage. These results highlight the biosensor's potential as a rapid, sensitive, and reproducible diagnostic platform for early HIV detection.

**Supplementary Information:**

The online version contains supplementary material available at 10.1007/s13205-025-04400-8.

## Introduction

In recent years, the advancement of nanotechnology has revolutionized the field of biosensor technology by offering remarkable opportunities for the development of sensitive and specific diagnostic platforms for various diseases. Human Immunodeficiency Virus (HIV) is still a global health challenge (Gayle and Hill 2001). HIV infects and progressively weakens the human immune system; untreated HIV leads to Acquired Immune Deficiency Syndrome (AIDS) (Mignogna and Lay 2011). Therefore, early diagnosis and timely initiation of antiretroviral therapy are crucial in managing HIV infection effectively (Girma et al. 2025). Because immunoglobulins appear only weeks after infection, among the various HIV envelope proteins, gp41 has been identified as a biomarker for early diagnosis due to its prominent role in viral entry (Arrildt et al. 2012). gp41 is primarily located within the viral envelope; however, when gp120 binds to a CD4 receptor, gp120 changes its conformation, which exposes gp41 (Chan et al. 1997; Guttman et al. 2012; Elalouf et al. 2024). While gp120 mediates viral attachment to CD4 receptors on host cells, gp41 is responsible for facilitating viral fusion and entry into the target cell. The gp41 protein possesses several unique characteristics that make it an attractive target for early HIV diagnosis (Yu et al. 2013). Firstly, gp41 is highly conserved among different HIV strains, making it a reliable biomarker for detection across various viral subtypes. Its preservation across strains ensures diagnostic accuracy and reproducibility, irrespective of the geographic distribution of HIV. Secondly, during viral fusion, gp41 undergoes conformational changes, leading to exposure of conserved regions known as the heptad repeat domains (HR1 and HR2) (Mische et al. 2005). These regions are involved in the formation of a stable six-helix bundle (6-HB) structure, which is essential for viral entry into host cells. Therefore, the presence of these conserved regions makes gp41 an ideal target for the development of specific diagnostic assays. gp41 is present on the viral surface, making it easily accessible for recognition by antibodies and other detection molecules. This accessibility contributes to the sensitivity of diagnostic assays that target gp41, enabling early detection of infection even at low viral loads (Joshi et al. 2020). Therefore, gp41, a critical envelope protein of HIV, has emerged as a crucial target for early diagnosis due to its prominent role in viral entry and fusion with host cells and the conserved nature of gp41, along with its accessibility on the viral surface, makes it an ideal biomarker for sensitive and specific diagnostic assays. In this study, we present the gp41 biosensor by highlighting the advantages of the nanomaterials used and their impact on overall performance. The novelty is to reduce the possible denaturation of the biorecognition receptor on biosensors by using peptides. Peptides are less immunogenic than antibodies and less prone to denaturation than aptamers (Navien et al. 2021). The proposed biosensor offers not only enhanced sensitivity but also robust performance for rapid, label-free detection of gp41, making it a promising platform for early-stage HIV diagnosis and disease monitoring. The incorporation of polystyrene and gold nanospheres into the biosensor design significantly improves surface stability and signal transduction, addressing the critical need for reliable detection of the HIV envelope protein gp41. By advancing the integration of nanomaterials with electrochemical sensing, this work contributes meaningfully to global efforts in HIV diagnostics and supports the development of next-generation tools for infectious disease detection. The peptide targeting the protein gp41 is Enfuvirtide (Tsibris and Hirsch 2014) (ENF, T-20, Fuzeon, DP-178) (YTSLIHSLIEESQNQQEKNEQELLELDKWASLWNWF, IC50: 0.006 µg/ml) (https://dbaasp.org/). Enfuvirtide binds specifically to the hydrophobic N-terminal heptad repeat (NHR) region of the gp41 trimer, preventing the formation of the critical six-helix bundle (6-HB) structure and thereby inhibiting viral membrane fusion (Yao et al. 2012). This mechanism underpins its established role as an HIV entry inhibitor. In the context of biosensing, Enfuvirtide was selected as the biorecognition element due to its high specificity and affinity for gp41, functioning effectively as a ligand for target recognition. Unlike conventional antibodies, peptides such as Enfuvirtide offer enhanced stability, reduced denaturation risk, and compatibility with surface immobilization strategies, making them particularly advantageous for electrochemical biosensor applications (Uygun and Tasoglu 2024). Traditional biosensors often suffer from limited sensitivity and bioreceptor instability, especially when detecting low-abundance biomarkers like gp41. By integrating Enfuvirtide with a stable polystyrene–gold nanoparticle platform, this biosensor addresses these limitations and provides a robust, selective, and durable solution for early HIV detection.

## Materials and method

### Materials

This study was approved by the Kafkas University Medical Faculty Ethical Committee (28.04.2021; 64-2021/05). Real samples were collected from individuals to obtain serum as matrix and stored at – 80 °C until use. Samples were collected from the urology department. All chemicals and peptide sequences (HS-(CH_2_)_5_-YTSLIHSLIEESQNQQEKNEQELLELDKWASLWNWF) were obtained from Sigma Aldrich (USA). Carbon screen-printed electrodes were used as working electrode (C110) Metrohm Dropsens Spain. HIV-1 gp41 16kDa Recombinant Protein (RPPB5576) was obtained from MyBiosource. Electrochemical measurements were carried out with a PalmSens 3 potentiostat controlled by PSTrace 5.9 software. SEM pictures obtained from COXEM EM-30 Plus (Korea). Python 3.8.20 was used for coding and data processing.

### Method

**Fig. 1:**
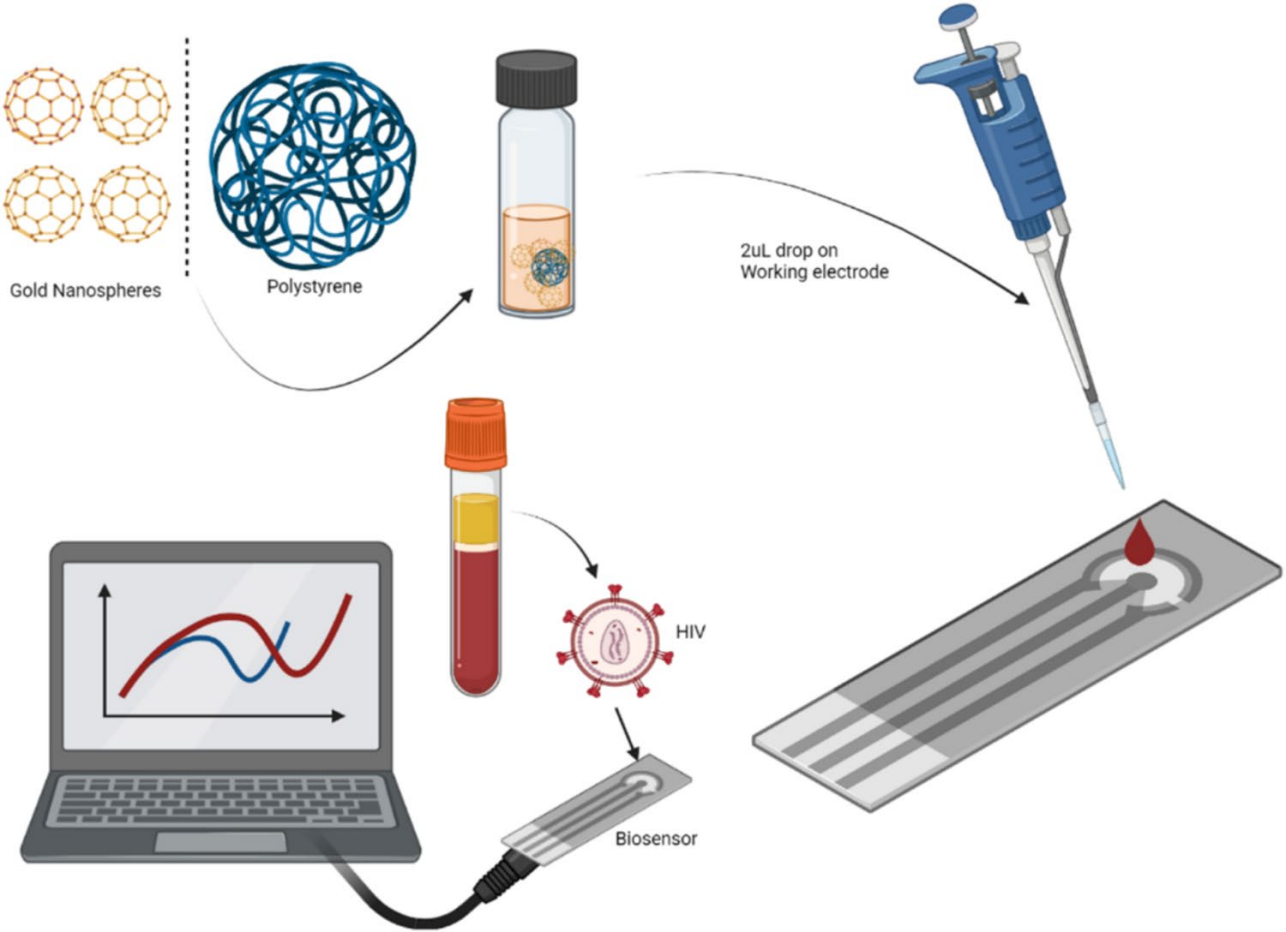
Schematic illustration of the fabrication process of the HIV gp41 impedimetric biosensor

Impedance methods were all collected and saved “*.csv file.” These files were processed step-by-step in Python, and the resulting data were used to train deep learning models (Suppl. File 1). We used a screen-printed carbon electrode (CE) incorporating a three-electrode system—carbon working and counter electrodes with an Ag/AgCl reference. EIS parameters were set as 10,000–0.05 Hz frequency by applying 0.18 V DC and 0.01V AC potentials (Onur Uygun and Ertuğrul Uygun 2020). EIS was performed in the redox probe solution of 5mM Fe(CN)_6_^4−/3−^ (Uygun et al. 2018). Prior to use electrodes were washed with excessive pure water and electrochemically cleaned by soaking electrode in 100mM H_2_SO_4_ and performed cyclic voltammetry the parameters were – 1.2V to 0.2 V with 100mV/s scan rate until the voltammograms superimpose (Uygun et al. 2020). Then the electrode was washed and dried under nitrogen gas flow. EIS was performed to investigate the bare electrodes. Then polystyrene–gold modification was carried out by slightly modified by a method (Tian et al. 2010). In order to prevent the size of the PS, in a glass vial, 3 mg thiolated polystyrene was dissolved inside of the 3mL chloroform and mixed. Into this solution 1mg/mL total 3mL AuNPs were added and mixed with slow stirring for 60 min. After one hour, the chloroform phase was evaporated and dried composite was dissolved in 1mL methanol. PS-AuNPs composite was dropped on the electrode surface as 2uL. This step was repeated 3 times (Fig. 1). It is important that this step must be carried out carefully to prevent the fast evaporation of methanol. The electrode was dried in ambient temperature and EIS was performed. After the CE-PS-AuNPs modification 50uL of 2mg/mL AMP was dropped on the electrode surface and incubated for overnight at + 4 °C under a humidified environment and this step was also observed after the modification by EIS. All these steps were also monitored by scanning electron microscopy. Serum samples were collected in anticoagulant tubes and centrifuged for 10 min at 4000 rpm. The samples were then tested by autoanalyzer for total protein concentration and was found as 78 mg/mL. Total 2 mg gp41 protein was dissolved in serum sample and total protein concentration of the serum was diluted by using serum to 300, 150, and 5 pg/mL concentrations and stored – 80 °C until use. These real samples were used for the matrix effect, real sample analysis, repeatability, and selectivity studies. After the biosensor modifications, the biosensor was tested by using chronoimpedimetric (CI) detection to obtain gp41 detection time. CI detection was carried out in blank in buffer, serum, 10 pg/mL gp41 in serum samples. Chronoimpedance parameters were set as 100mV AC, 10mV AC, and 1000 Hz for 800 s. Afterward, eight biosensors were prepared for storage stability and four electrodes were kept in room temperature (R.T.), and the others were kept in + 4 °C humidity and dark. 50 pg/mL real samples were measured with these biosensors and signal decrease compared with *t* = 0.

## Results and discussion

This biosensor was engineered by using a different method to increase the stability of the gold nanoparticles and increase the durability of the biosensor performance. Therefore, we used a thiolated-polystyrene layer to minimize hydrophilic interactions and reduce nonspecific adsorption and also to increase storage stability. Gold nanospheres were chosen for the self-assembly immobilization of the AMPs to surface. The structure also provided more sensitive structure because of the gold accumulation of the surface. The following impedance data and plots were generated from Python analysis. The model impedance spectra were trained and analyzed using equivalent circuit models (Uygun and Ertuǧrul Uygun 2014). The circuit model was chosen because of the obtained EIS curves, and the microscopy results showed quite good homogeneous layer with semi-circle; therefore, capacitance (*C*) was parallel to electrode surface transfer resistance (*R*2), which is the signal for our biosensor response for gp41; linear region of the EIS curve that serial to *R*2 was Warburg impedance (W); and *R*1 is serial to these parameters as solution resistance of the redox probe. In order to find out the biosensor response time (detection time), chronoimpedance, which measures impedance over time, was used to analyze dynamic interaction in how biomolecules interact with the biosensor surface (Onur and Akçay 2022). Deep learning models, especially neural networks, can detect nonlinear relationships that exist between frequency, impedance, and electrochemical parameters (Conlin et al. 2021). Manually extracting an appropriate equivalent circuit model from such interactions is time consuming. The model, once trained, can rapidly analyze large volumes of data without manual tuning. Analyzing these time-series data with deep learning methods, particularly Recurrent Neural Networks (RNNs) like LSTM (Long Short-Term Memory), offers a powerful alternative to more traditional analytical approaches for our biosensor project (Sherstinsky 2020; Dai et al. 2024). All these components can analyze whole data in minutes as given in the supplementary file (suppl. file).

**Fig. 2:**
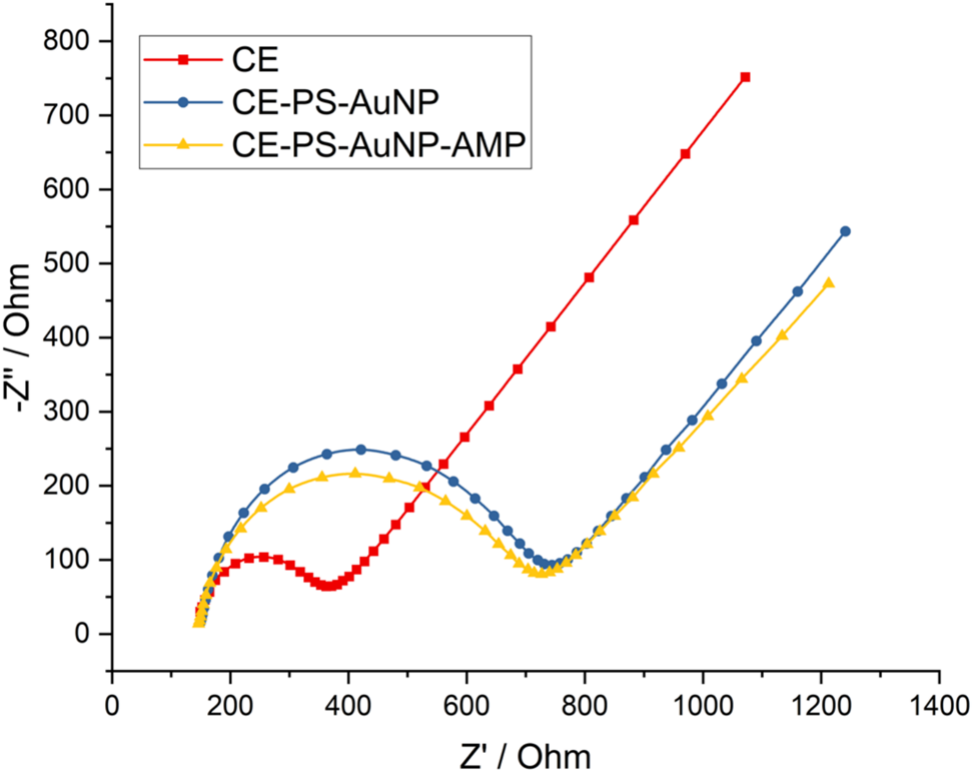
Electrochemical impedance spectroscopy (EIS) results showing biosensor surface modification. Nyquist plots represent the impedance behavior of (red) the bare carbon electrode, (blue) the electrode modified with PS-AuNP composite, and (yellow) the AMP-functionalized biosensor. The increasing diameter of semicircles corresponds to increasing charge transfer resistance (Rct), confirming successful surface functionalization at each stage

**Fig. 3:**
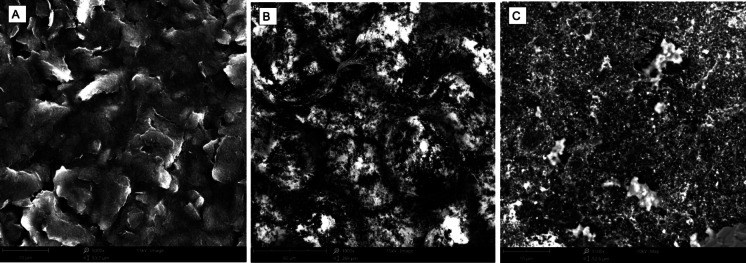
Surface morphology of the biosensor at different modification stages, characterized by SEM. **A** Bare carbon electrode. **B** Electrode modified with polystyrene–gold nanoparticles at 1000 × magnification, showing a homogeneous nanoparticle layer. **C** Electrode at 5000 × magnification, revealing dispersed gold nanoparticles embedded in the polystyrene matrix, providing anchoring sites for peptide immobilization

Experimentally, electrode surface modifications were observed electrochemically by EIS in Fig. 2 and performed by CV in Supplementary Fig. 1. The EIS data reveal the stepwise modification of the electrode surface. The red curve corresponds to the bare carbon electrode, the blue curve represents the polystyrene–gold (PS-AuNP) modified electrode, and the yellow curve indicates the AMP-immobilized surface. As shown in the curves, surface modifications led to a progressive increase in charge transfer resistance (Rct), with the exception of the AMP-functionalized electrode, where a relative decrease was observed. This resistance, derived from EIS under a frequency-dependent potential, reflects the hindrance of electron transfer between the redox probe and the electrode surface. AMPs (antimicrobial peptides) possess positively charged functional groups that electrostatically attract the negatively charged ferri/ferrocyanide redox species, increasing their local concentration at the electrode interface. This enhanced local availability of the redox probe contributes to improved electron transfer and a reduction in impedance, as observed after AMP immobilization. The typical Nyquist plots exhibit a semicircular region at high frequencies, representing charge transfer resistance, followed by a linear Warburg region at low frequencies, which indicates diffusion-limited mass transport. Figure 3 provides supporting SEM images: (A) shows the unmodified carbon electrode; (B) and (C) display the polystyrene–gold nanoparticle-modified surfaces at increasing magnifications. The images reveal bright, granular features corresponding to gold nanoparticles embedded within the polystyrene matrix, which serve as anchoring sites for peptide immobilization. This high-resolution SEM image shows the unmodified screen-printed carbon electrode (SPCE). The surface appears rough, flaky, and heterogeneous, which is characteristic of carbon-based working electrodes. This topography provides a high surface area that can facilitate the adsorption and immobilization of nanomaterials or biorecognition molecules in subsequent modification steps. This lower magnification image reveals macro-scale distribution of polystyrene–gold composites across the electrode surface. Distinct bright spherical and irregular clusters are observed, which correspond to aggregated polystyrene matrices embedded with gold nanoparticles. The uneven distribution is typical of drop-casted films, especially with hydrophobic components like polystyrene. These composite features provide a hydrophobic barrier while simultaneously acting as anchoring sites for peptide immobilization through thiol–gold interactions. The textured surface also enhances electron transfer dynamics via gold’s conductivity. At this visual (C), the image reveals a dense, granular nanostructure, representing the final surface after AMP (Enfuvirtide) peptide immobilization onto the PS-AuNP-modified electrode. The bright, scattered nanoparticulate features suggest a successful immobilization of peptides, possibly due to clustering on gold-rich regions. The matrix appears more homogeneous and granular, which reflects a well-distributed peptide layer over the gold–polystyrene scaffold. This morphology is optimal for biosensing, as it supports stable and accessible biorecognition sites for the target gp41 protein.

**Fig. 4:**
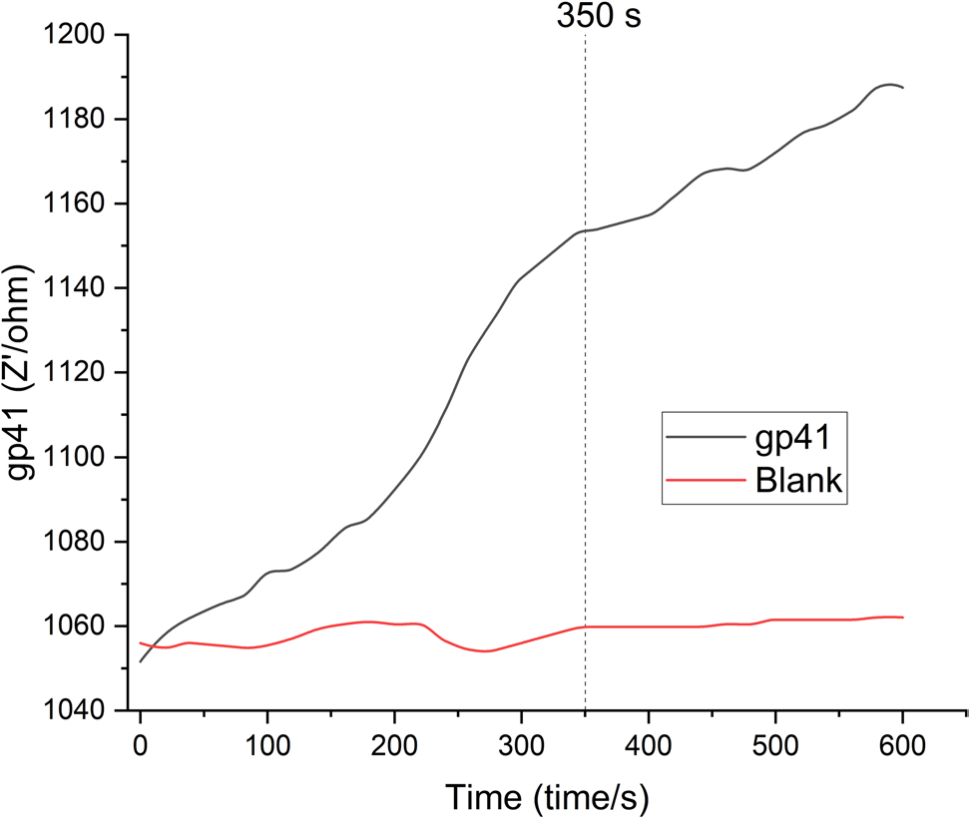
Chronoimpedimetric detection of HIV gp41 protein in serum. The plot shows real-time impedance changes over 800 s using biosensors in (red) blank buffer and (black) serum spiked with 80 pg/mL gp41. A significant rise in impedance is observed in the gp41-spiked sample around 350 s, indicating successful binding and detection

After the modification of the electrodes, gp41 detection time was optimized by the chronoimpedimetric detection shown in Fig. 4 and performed in buffer (red CI) and gp41 (black CI) included serum sample. As shown in Fig. 4, gp41 binds to surface in 350 s, distinguishably. Even the first seconds seem too good for the detection time; y-axis shows the impedance increase is slow. Slow increase is because of the hydrophobic structure of the biosensor, and this shows an advantage by preventing the adsorption (red CI). Although slight impedance changes are observed earlier, the initial signal increase is gradual. This delayed and controlled signal development is attributed to the hydrophobic nature of the polystyrene-based sensor surface, which effectively minimizes nonspecific adsorption. This property ensures that the impedance increase observed is due primarily to specific biomolecular interactions rather than background noise or matrix effects, thus enhancing the selectivity and reliability of the biosensor.

**Fig. 5:**
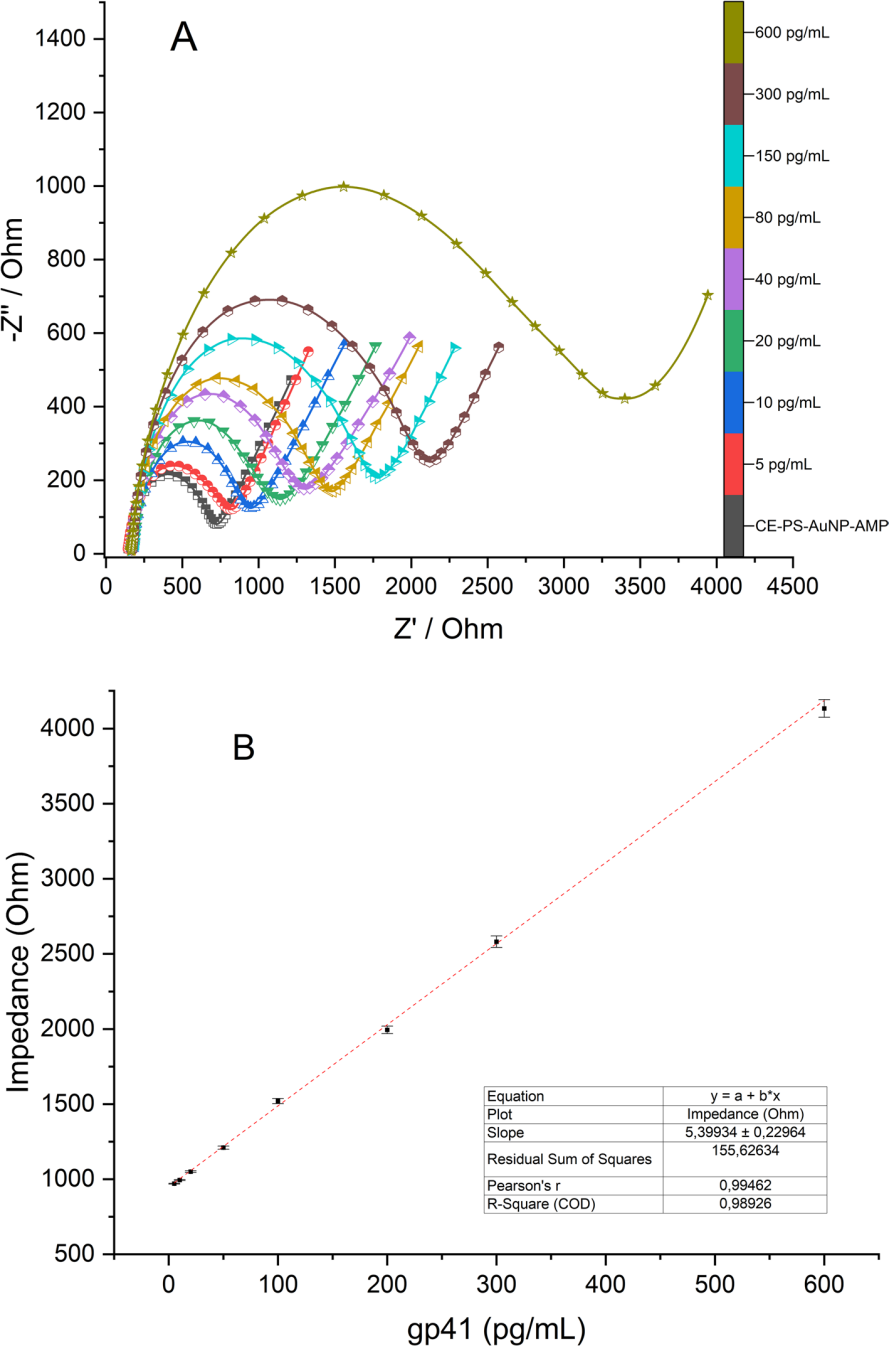
Calibration performance of the biosensor for HIV gp41 detection. **A** EIS spectra collected after exposure to increasing concentrations of gp41 (5–600 pg/mL). **B** Calibration curve based on the fitted impedance values (Rct) versus concentration, showing excellent linearity (*R*^2^ = 0.9946). The detection limit (LOD) and quantification limit (LOQ) were calculated as 1.62 pg/mL and 4.91 pg/mL, respectively

The biosensor calibration curve was prepared by incubating the gp41 protein solution on the biosensor and incubated for ~ 6 min. After every six minutes biosensor surface was measured by EIS shown in Fig. 5A. Linearity was observed between 5 and 600 pg/mL and we prepared six different biosensors for the assessment of the biosensor reproducibility and R^2^ was found as 0.9946 ± 0.0022 (CV%: 0.22%). This shows quite good reproducibility because of the stabile structure of the biosensor. The data obtained in Fig. 5A, calculated by fitting the EIS curves for the circuit model that is shown in supplementary file. This circuit model is the Randles circuit model that includes R1 and R2 as redox solution and working electrode impedances, respectively. W represents the mass transfer resistance of Warburg impedance and Cdl shows the surface double layer capacitance. The R2 values give the EIS data by comparing the concentrations the gp41 (Fig. 5B). Impedance data fitting and R2 calculation were carried out by Python programming. First, Python NumPy, matplotlib, and curve_fitting were used as library for fitting. After the installation Terminal/command prompt/Git BASH code was used to update. The following codes were formed for fitting. According to the calibration curve LOD and LOQ were calculated as 1.62 pg/mL and 4.91 pg/mL, respectively. LOQ is very close to the calibration curve beginning point that shows the calibration curve is mathematically good. The lowest concentrations were reached by the AMPs for the gp41 proteins. As can be seen, their IC50 values are very low; in other words, the affinity for the gp41 is very high.

**Fig. 6:**
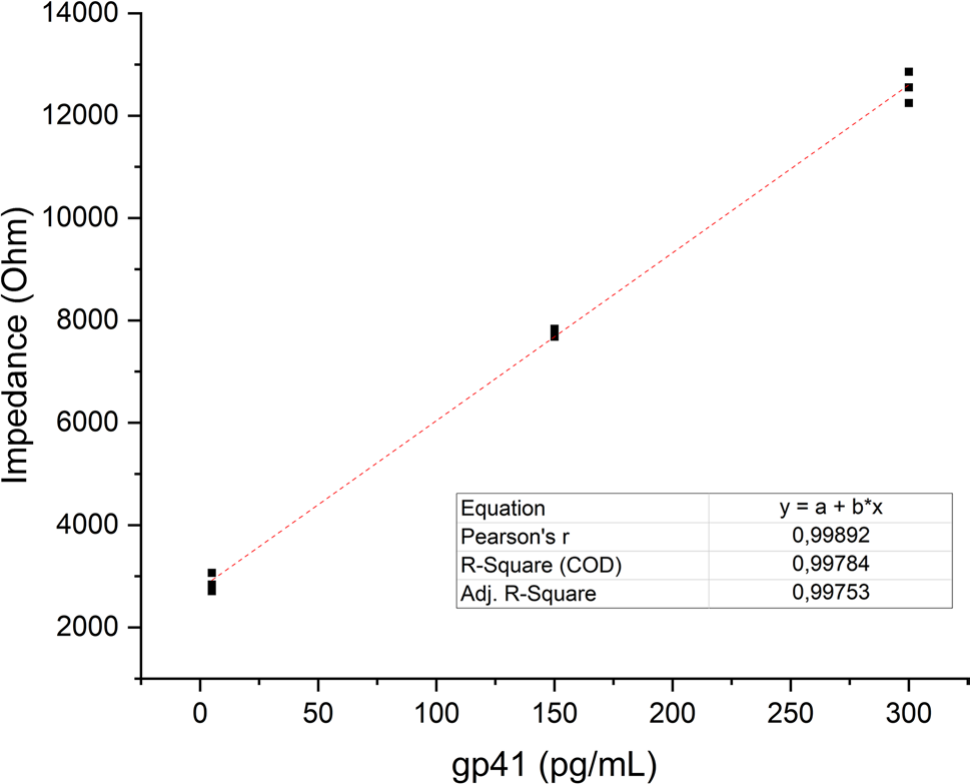
Biosensor performance in real serum samples: selectivity and repeatability. Biosensors were tested with varying gp41 concentrations (including low and high concentrations within the calibration range). The results demonstrate consistent response across multiple sensors and minimal matrix effect, confirming the biosensor’s robustness in complex biological samples

**Fig. 7:**
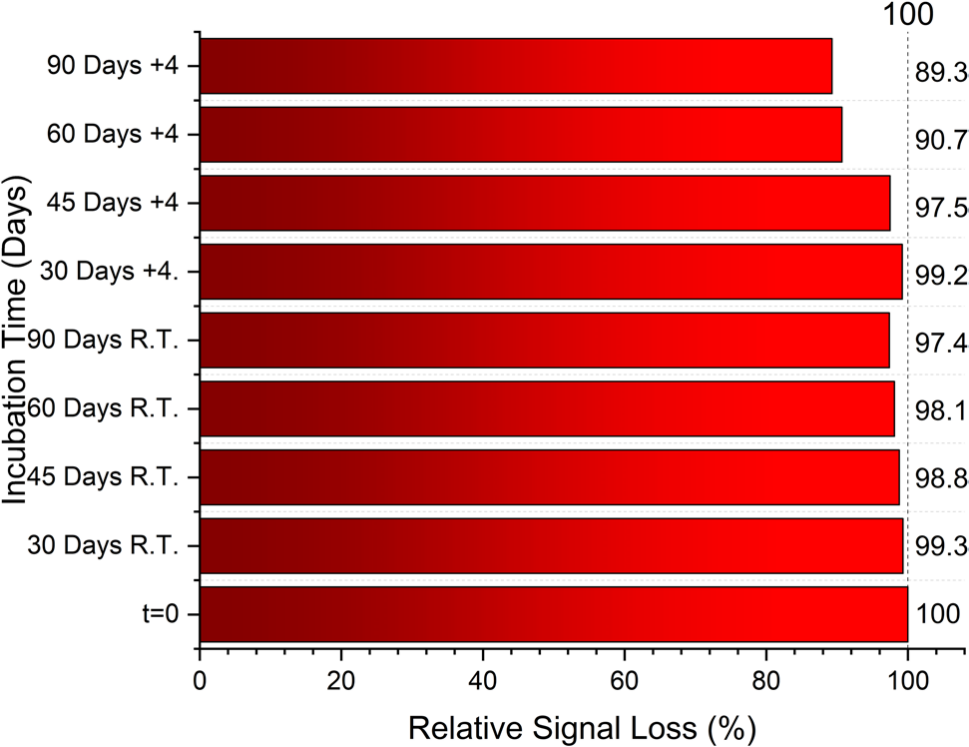
Storage stability of the biosensor under different conditions. Biosensors stored at room temperature (R.T.) and + 4 °C were tested after a defined period using a 50 pg/mL gp41-spiked sample. The performance was compared to freshly prepared sensors (*t* = 0). The biosensors stored at room temperature exhibited better long-term stability, likely due to the hydrophobic nature of the polystyrene–gold matrix

In Fig. 6, electrode selectivity, repeatability, and real sample detection were tested by high concentrations and the lowest concentrations of the calibration curve of EIS (Suppl. Figure 2). Already diluted samples were applied for different biosensors and tested. Each concentration is represented by three close EIS curves, confirming high reproducibility in the biosensor response. Even at 5 pg/mL, the system exhibits a distinct EIS, showing that the biosensor can detect low levels of gp41 reliably. The biosensor shows good response for selectivity and sensitivity. Repeating the samples with different electrodes shows good correlation. This is provided by the stabile surface modification technique and represents the immobilization provides equally binding sites in every modification step. Selectivity of the real samples shows not more than 12% signal differences, which is quite good for the biosensor system. Final optimization of the biosensor was storage stability and biosensor shows good stability against the time. However, room storage kept stable the styrene, where gold inside it, more than cool temperature, which increased the storage stability shown in Fig. 7. Polystyrene (PS) is a hydrophobic thermoplastic polymer (Zimmermann 2021). At low temperatures, PS can become more brittle or contract slightly, potentially affecting the uniform distribution of gold nanoparticles embedded within it. PS shrinks or stiffens, which may lead to cracking or delamination at the biosensor interface, impairing performance. Cold storage could suppress this flexibility and promote unwanted conformational changes or moisture absorption over time. In conclusion, the biosensors showed good performance by comparing other biosensors (Table 1).

**Table 1:** Comparison of gp41 biosensor performances

Method	Modification	LOD	Linear Range	Detection Time	References
Electrochemical	PS-AuNPs-AMP	1.62 pg/mL	5–600 pg/mL	350 Seconds	This study
Electrochemical	Gold-Metallopeptide	1.5 nM	–	–	(Hou and Gochin 2008)
Optical	APTMS-anti-gp41	25 ng/mL	–	10–12 Minutes	(Manoto et al. 2020)
Electrochemical	Anti-gp41immuno-membrane; Mimic peptide (EGIEE)	–	–	10–15 min	(Uda et al. 1995)
Piezoelectric	Epitope-imprinted polymer	2 ng/mL	5–200 ng/mL	minutes	(Lu et al. 2012)

*EIS* electrochemical impedance spectroscopy, *CV* cyclic voltammetry, *SPR* surface plasmon resonance, *PS* polystyrene, *AuNP* gold nanoparticles, *APTMS* (3-aminopropyl) trimethoxysilane, *QCM* quartz crystal microbalance, *MIP* molecularly imprinted polymer, *ELISA* enzyme-linked immunosorbent assay, *MPER* membrane-proximal external region of gp41, *SAW* surface acoustic wave, *SH-SAW* shear horizontal SAW, *FET* field-effect transistor

## Conclusion

In this study, we report for the first time in literature a hydrophobic, antimicrobial peptide-based biosensor for the label-free detection of the HIV envelope protein gp41. The biosensor was constructed by modifying a carbon electrode with a polystyrene–gold nanoparticle (PS-AuNP) composite, providing a water-resistant interface that supports stable peptide immobilization. The use of electrochemical impedance spectroscopy (EIS) enabled highly sensitive detection without the need for labeling. Compared to traditional biosensors, the signal increase in this platform is more controlled due to the hydrophobic surface, which effectively minimized nonspecific adsorption from serum components. This property not only enhanced specificity but also contributed to a clearer, more reliable analytical signal. The biosensor demonstrated excellent linearity, reproducibility, and selectivity across a clinically relevant concentration range of gp41. Importantly, all electrochemical data were analyzed using Python-based algorithms, enabling rapid and automated processing within minutes, significantly reducing the overall detection time. Taken together, this biosensor platform offers a promising and scalable strategy for early HIV diagnosis and lays the foundation for future developments in nanomaterial-integrated biosensing technologies.

## Supplementary Information

Below is the link to the electronic supplementary material.

Supplementary file1 (DOCX 404 KB)

## Data Availability

The datasets generated and/or analyzed during the current study are available from the corresponding author upon reasonable request.
