# Supplementary material for: Python-driven impedance profiling on peptide-functionalized biosensor for detection of HIV gp41 envelope protein
Source: 3 Biotech. 2025 Jun 30;15(7):229. doi: 10.1007/s13205-025-04400-8 (PMC12209138; doi:10.1007/s13205-025-04400-8)
Supplement: Supplementary file 1 — Supplementary file1 (DOCX 404 KB) [file 13205_2025_4400_MOESM1_ESM.docx]

**Supplementary File**


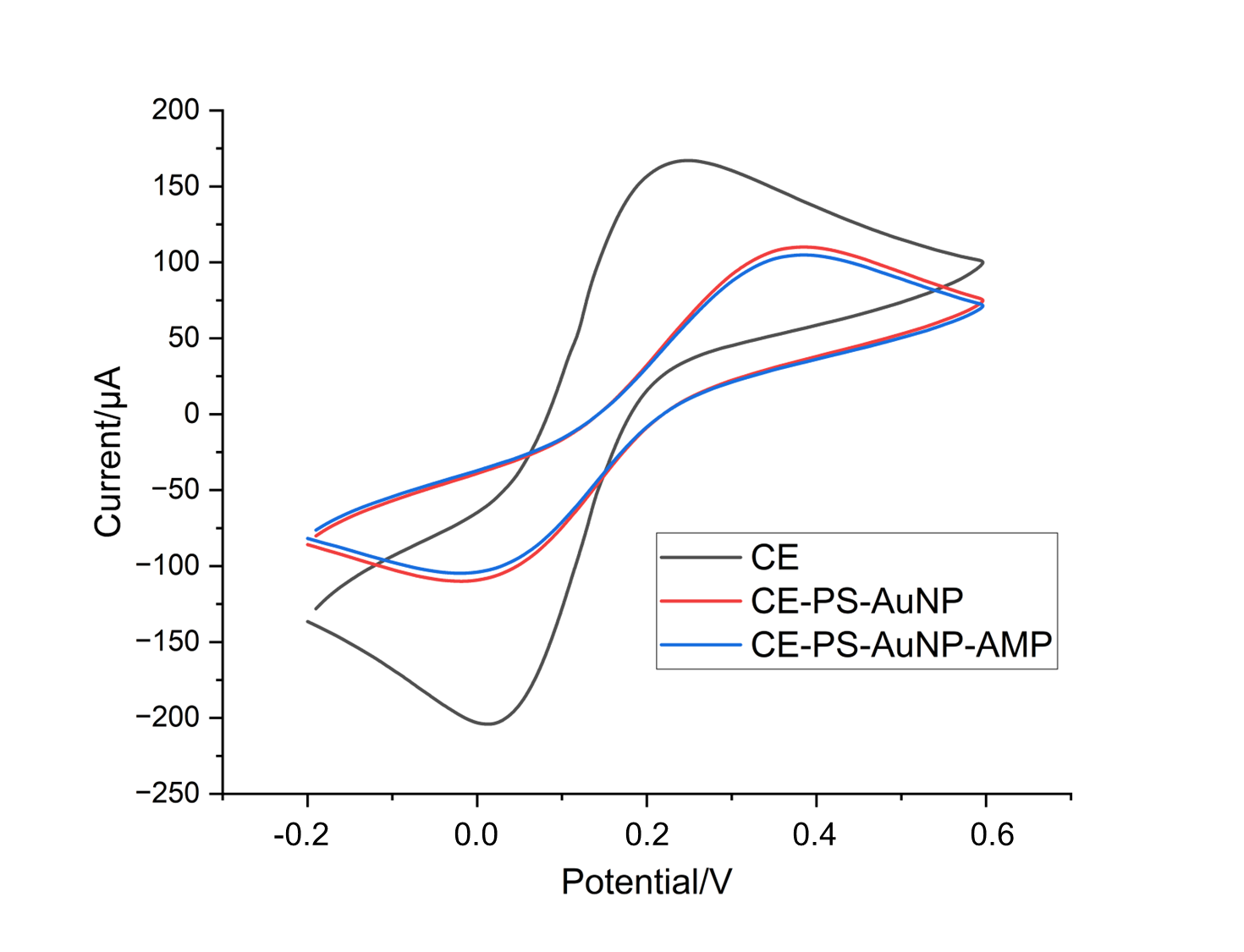


Supplementary figure 1. Biosensor modifications by CV curves.


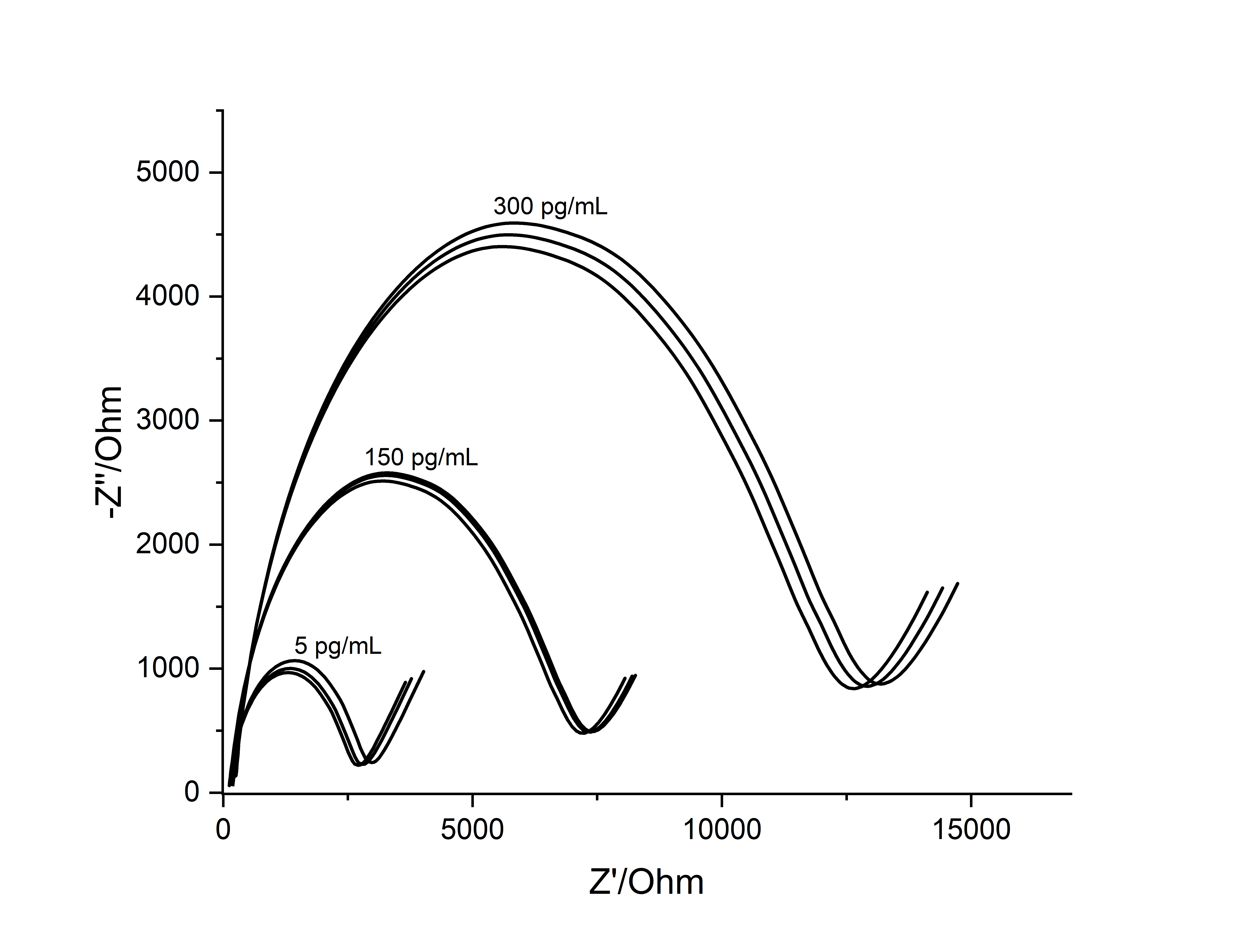


Supplementary figure 2. EIS spectrums of the real samples of figure 6.

1. **Python codes for data analysis**
   1. **Impedance Data Fitting Using Equivalent Circuit Models**

import numpy as np

from scipy.optimize import curve_fit

import matplotlib.pyplot as plt

# Define the Randles circuit model function

def randles_impedance(frequency, R1, R2, Cdl, W):

omega = 2 * np.pi * frequency

Z_R1 = R1

Z_R2 = R2

Z_Cdl = 1 / (1j * omega * Cdl)

Z_W = W / (np.sqrt(1j * omega))

Z_total = Z_R1 + (1 / ((1 / Z_R2) + (1 / Z_Cdl) + (1 / Z_W)))

return Z_total.real, -Z_total.imag # Returning real and imaginary parts

# Load experimental frequency and impedance data

frequency_data = np.loadtxt('frequency_data.txt') # replace with actual frequency data file

impedance_real_data = np.loadtxt('impedance_real.txt') # real part

impedance_imag_data = np.loadtxt('impedance_imag.txt') # imaginary part

# Combine real and imaginary parts into a single dataset for curve fitting

def combined_impedance_model(frequency, R1, R2, Cdl, W):

Z_real, Z_imag = randles_impedance(frequency, R1, R2, Cdl, W)

return np.concatenate([Z_real, Z_imag])

# Initial guesses for the parameters R1, R2, Cdl, and W

initial_guesses = [100, 200, 1e-6, 10]

# Perform curve fitting

popt, _ = curve_fit(combined_impedance_model, frequency_data, np.concatenate([impedance_real_data, impedance_imag_data]), p0=initial_guesses)

R1, R2, Cdl, W = popt

print("Fitted Parameters:")

print(f"R1: {R1}, R2: {R2}, Cdl: {Cdl}, W: {W}")

# Plot the fitted vs actual impedance data

fitted_real, fitted_imag = randles_impedance(frequency_data, *popt)

plt.figure()

plt.plot(frequency_data, impedance_real_data, 'o', label="Real Data")

plt.plot(frequency_data, fitted_real, '-', label="Fitted Real")

plt.plot(frequency_data, -impedance_imag_data, 'o', label="Imag Data")

plt.plot(frequency_data, -fitted_imag, '-', label="Fitted Imag")

plt.xlabel("Frequency (Hz)")

plt.ylabel("Impedance (Ohm)")

plt.legend()

plt.show()

***Circuit Model of the Biosensor***

R1

|

|

-------------------

| |

| |

R2 W

| |

| |

Cdl

|

|

---

-

- 1. **Chronoimpedance Detection for gp41 Concentration Analysisimport pandas as pd**

import matplotlib.pyplot as plt

# Load chronoimpedance data

# Assume data file format: time (s), impedance (ohm) columns

data = pd.read_csv('chronoimpedance_data.csv') # replace with actual data file

# Plot chronoimpedance data

plt.figure()

plt.plot(data['time'], data['impedance'], label="Impedance over Time")

plt.xlabel("Time (s)")

plt.ylabel("Impedance (Ohm)")

plt.title("Chronoimpedance Detection of gp41")

plt.legend()

plt.show()

# Extract steady-state impedance values and calculate calibration curve

concentrations = [5, 10, 20, 50, 100, 200, 300, 600] # example concentrations in pg/mL

steady_state_impedance = [data[data['time'] > 600]['impedance'].mean() for conc in concentrations]

plt.figure()

plt.plot(concentrations, steady_state_impedance, 'o-', label="Calibration Curve")

plt.xlabel("gp41 Concentration (pg/mL)")

plt.ylabel("Steady-State Impedance (Ohm)")

plt.title("Calibration Curve for gp41 Detection")

plt.legend()

plt.show()

- 1. **Limit of Detection (LOD) and Limit of Quantification (LOQ) Calculation**

# Calculate the slope and intercept of the calibration curve

from scipy.stats import linregress

slope, intercept, _, _, std_err = linregress(concentrations, steady_state_impedance)

LOD = 3.3 * std_err / slope

LOQ = 10 * std_err / slope

print(f"Limit of Detection (LOD): {LOD:.2f} pg/mL")

print(f"Limit of Quantification (LOQ): {LOQ:.2f} pg/mL")

1. **Deep Learnin Model for Data Analysis**
   1. **Deep Learning Model for Impedance Spectra Fitting**

import numpy as np

import tensorflow as tf

from tensorflow.keras.models import Sequential

from tensorflow.keras.layers import Dense

from sklearn.model_selection import train_test_split

# Generate or load impedance data for training (frequency, R1, R2, Cdl, W, impedance_real, impedance_imag)

frequency_data = np.load('frequency_data.npy')

params_data = np.load('params_data.npy') # R1, R2, Cdl, W parameters

impedance_real_data = np.load('impedance_real.npy')

impedance_imag_data = np.load('impedance_imag.npy')

# Prepare input and output data for the neural network

X = np.column_stack((frequency_data, params_data)) # Inputs: frequency and Randles parameters

Y_real = impedance_real_data # Real part of impedance

Y_imag = impedance_imag_data # Imaginary part of impedance

# Split data into training and test sets

X_train, X_test, Y_real_train, Y_real_test = train_test_split(X, Y_real, test_size=0.2, random_state=42)

_, _, Y_imag_train, Y_imag_test = train_test_split(X, Y_imag, test_size=0.2, random_state=42)

# Define the neural network model

model_real = Sequential([

Dense(64, input_shape=(X.shape[1],), activation='relu'),

Dense(64, activation='relu'),

Dense(1) # Predict real part of impedance

])

model_imag = Sequential([

Dense(64, input_shape=(X.shape[1],), activation='relu'),

Dense(64, activation='relu'),

Dense(1) # Predict imaginary part of impedance

])

# Compile the models

model_real.compile(optimizer='adam', loss='mean_squared_error')

model_imag.compile(optimizer='adam', loss='mean_squared_error')

# Train the models

model_real.fit(X_train, Y_real_train, epochs=100, validation_data=(X_test, Y_real_test), batch_size=32)

model_imag.fit(X_train, Y_imag_train, epochs=100, validation_data=(X_test, Y_imag_test), batch_size=32)

# Predict impedance for test data

real_pred = model_real.predict(X_test)

imag_pred = model_imag.predict(X_test)

# Plot predictions vs actual impedance values

import matplotlib.pyplot as plt

plt.figure()

plt.scatter(Y_real_test, real_pred, label='Real Impedance Prediction')

plt.xlabel("Actual Real Impedance")

plt.ylabel("Predicted Real Impedance")

plt.legend()

plt.show()

plt.figure()

plt.scatter(Y_imag_test, imag_pred, label='Imaginary Impedance Prediction')

plt.xlabel("Actual Imaginary Impedance")

plt.ylabel("Predicted Imaginary Impedance")

plt.legend()

plt.show()

- 1. **Deep Learning Model for Chronoimpedance Analysis**

from tensorflow.keras.layers import LSTM

from tensorflow.keras.models import Sequential

from tensorflow.keras.layers import Dense

# Load or generate chronoimpedance time-series data for training

# X_chrono: input data with shape (samples, timesteps, features)

# Y_concentration: target gp41 concentration

X_chrono = np.load('chronoimpedance_data.npy') # Assume shape (samples, timesteps, features)

Y_concentration = np.load('concentration_data.npy') # Corresponding gp41 concentrations

# Split the data into training and test sets

X_chrono_train, X_chrono_test, Y_concentration_train, Y_concentration_test = train_test_split(

X_chrono, Y_concentration, test_size=0.2, random_state=42

)

# Define an LSTM model for time-series prediction

chrono_model = Sequential([

LSTM(64, input_shape=(X_chrono.shape[1], X_chrono.shape[2]), activation='relu'),

Dense(64, activation='relu'),

Dense(1) # Output: gp41 concentration

])

# Compile the model

chrono_model.compile(optimizer='adam', loss='mean_squared_error')

# Train the model

chrono_model.fit(X_chrono_train, Y_concentration_train, epochs=50, batch_size=16, validation_data=(X_chrono_test, Y_concentration_test))

# Predict concentrations for test data

concentration_pred = chrono_model.predict(X_chrono_test)

# Plot predictions vs actual concentrations

plt.figure()

plt.scatter(Y_concentration_test, concentration_pred, label="Predicted vs Actual gp41 Concentration")

plt.xlabel("Actual Concentration (pg/mL)")

plt.ylabel("Predicted Concentration (pg/mL)")

plt.legend()

plt.show()
